# Discovery of Hybrid Dual *N-*Acylhydrazone and Diaryl Urea Derivatives as Potent Antitumor Agents: Design, Synthesis and Cytotoxicity Evaluation

**DOI:** 10.3390/molecules18032904

**Published:** 2013-03-04

**Authors:** Xin Zhai, Qiang Huang, Nan Jiang, Di Wu, Hongyu Zhou, Ping Gong

**Affiliations:** Key Laboratory of Structure-Based Drug Design & Discovery, Ministry of Education, School of Pharmaceutical Engineering, Shenyang Pharmaceutical University, Shenyang 110016, Liaoning, China

**Keywords:** diaryl ureas, *N*-acylhydrazone, cytotoxicity

## Abstract

Based on the hybrid pharmacophore design concept, a novel series of dual diaryl urea and *N*-acylhydrazone derivatives were synthesized and evaluated for their *in vitro* cytotoxicity by the standard MTT assay. The pharmacological results indicated that most compounds exhibited moderate to excellent activity. Moreover, compound **2g** showed the most potent cytotoxicity against HL-60, A549 and MDA-MB-231 cell lines, with IC_50_ values of 0.22, 0.34 and 0.41 μM, respectively, which was 3.8 to 22.5 times more active than the reference compounds sorafenib and PAC-1. The promising compound **2g** thus emerges as a lead for further structural modifications.

## 1. Introduction

**Figure 1:**
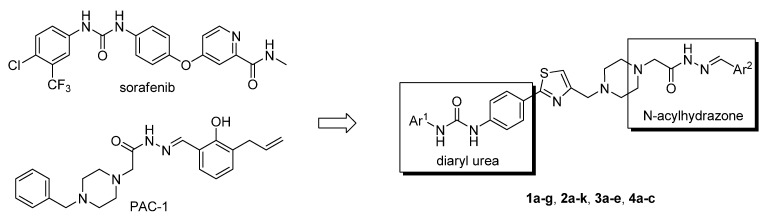
Structures of sorafenib, PAC-1 and target compounds.

Design of single chemical compounds that simultaneously modulate multiple biological targets in a specific manner is the current focus of new drug development and is becoming more popular. An effective approach is to take existing individual compounds, each known to have pharmacological structural features and high selectivity against the particular targets of interest, and combine them into a single molecule. Sorafenib (Figure 1), a diaryl urea analogue [1], is a small molecular inhibitor of several tyrosine protein kinases (VEGFR, PDGFR and B-Raf) [2,3] and unique in targeting the Raf/Mek/Erk pathway (MAPK pathway) [4], was approved by FDA for the treatment of advanced renal cell carcinoma and advanced hepatocellular carcinoma [5,6]. PAC-1 (Figure 1), the first preferential small molecule procaspase-3 activating compound with *N*-acylhydrazone pharmacophore, is promising as a new anti-tumor drug that can directly influence the apoptotic machinery or suicide of cells and has shown good results in mouse models [7,8,9].

In an attempt to discover new antitumor agents with multiple molecular mechanisms, we combined the diaryl urea moiety from sorafenib and *N*-acylhydrazone based in a hybrid pharmacophore design. The use of thiazolyl and amido moieties as linkers has been reported in our preliminary study [10], and the thiazolyl ring is retained in this paper for its better antitumor potency. Thus a series of diaryl urea derivatives bearing an *N*-acylhydrazone moiety (Figure 1) were designed and synthesized. Various substituted ureido-linked phenyl (Ar^1^) and hydrazone-linked phenyl (Ar^2^) groups were introduced to explore the influence of electronic and steric effects on the anticancer activity. 2-Hydroxyl substitution was retained for the Ar^2^ ring for the reason that only with the hydroxyl group on Ar^2^ did the PAC-1 derivatives display antitumor activity *in vitro *[11]. 4-and 5-Benzyloxyl groups were introduced to Ar^2^, respectively, to investigate the effect of the extension of the hydrophobic region. Furthermore, Ar^2^ was replaced with a substituted chromenonyl or imidazolindionyl groups, which are often associated with a variety of biological activity, to note the effect of each discreet change on the biological activity of the resulting compounds.

## 2. Results and Discussion

### 2.1. Chemistry

**Scheme 1:**
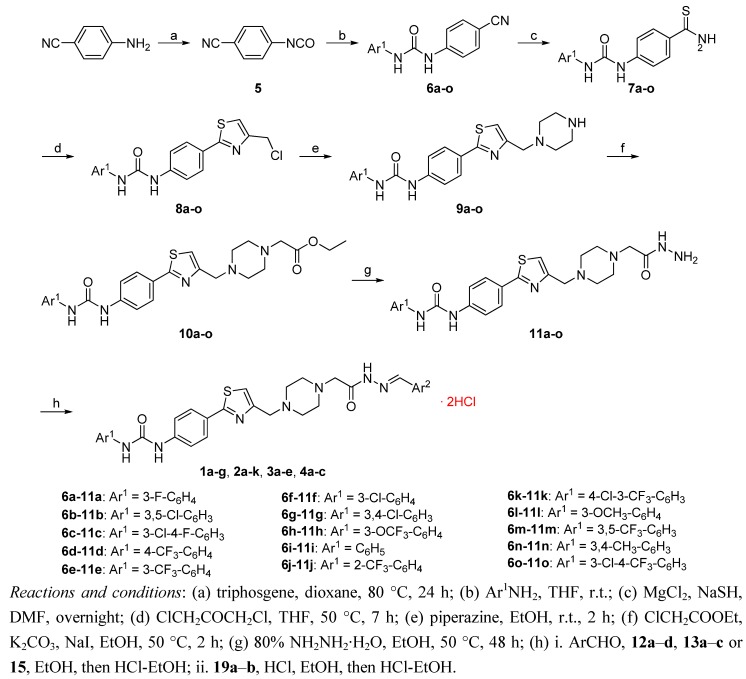
Synthesis of target compounds **1a**–**g**, **2a**–**k**, **3a**–**e** and **4a**–**c**.

The synthesis of target compounds **1a**–**g**, **2a**–**k**, **3a**–**e** and **4a**–**c** is described in Scheme 1. Commercially available 4-aminobenzonitrile reacted with triphosgene in dioxane at 80 °C for 24 h to give 4-isocyanatobenzonitrile (**5**) as a colorless oil. Compound **5** was treated with various substituted anilines to obtain diaryl ureas **6a**–**o** [12], whose cyano group was reduced to a thioamide moiety using magnesium chloride and sodium hydrogensulfide in *N*,*N*-dimethylformamide to afford the corresponding derivatives **7a**–**o**. Cyclization of **7a**–**o** with 1,3-dichloroacetone in tetrahydrofuran at 50 °C readily afforded thiozoles **8a**–**o**, which reacted with piperazine in ethanol by nucleophilic substitution to give **9a**–**o**. Consequently, treatment of **9a**–**o** with ethyl chloroacetate in ethanol in the presence of potassium carbonate and sodium iodide afforded esters **10a**–**o**, which were turned into acylhydrazines **11a**–**o** via hydrazinolysis in 80% hydrazine hydrate for 48 h. Finally, target compounds **1a**–**g**, **2a**–**k**, **3a**–**e** and **4a**–**c** were prepared via condensation of **11a**–**o** with substituted benzaldehydes, various aromatic aldehydes (**12a**–**d**, **13a**–**c** or **15**) as well as imidazolindiones **19a**–**b**, respectively, and isolated as the corresponding dihydrochlorides [7].

**Scheme 2:**
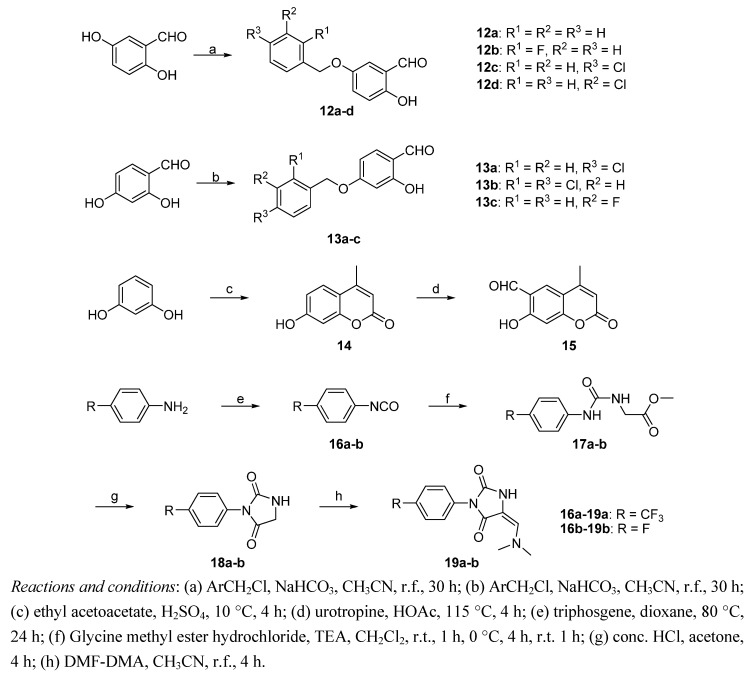
Synthesis of intermediates **12a**–**d**, **13a**–**c**, **15** and **19a**–**b**.

As shown in Scheme 2, aryloxybenzaldehydes **12a**–**d** and **13a**–**c** were prepared from 2,5-(or 2,4-) dihydroxybenzaldehyde via regioselective *O*-alkylation reactions with benzyl chlorides in acetonitrile in the presence of sodium hydrogencarbonate and potassium iodide. Cyclization of *m*-dihydroxy-benzene with ethyl acetoacetate in sulfuric acid at 10 °C, followed by formylation with urotropine in glacial acetic acid and sulfuric acid in sequence, namely a Duff reaction, provided chromenealdehyde **15** as a white solid [13]. Imidazolindiones **19a**–**b** were synthesized from substituted anilines, which were turned into phenyl isocyanates **16a**–**b** in a similar manner as described for compound **5**. Subsequent treatment of **16a**–**b** with glycine methyl ester hydrochloride in the presence of triethylamine in dichloromethane gave ureas **17a**–**b**. Cyclization of **17a**–**b** in the presence of concentrated hydrochloride gave rise to **18a**–**b**, which were further condensed with *N*,*N*-dimethylformamide dimethylacetal (DMF-DMA) to obtain **19a**–**b** [14].

### 2.2. Biological Results and Discussion

All target compounds **1a**–**g**, **2a**–**k**, **3a**–**e** and **4a**–**c** were evaluated for their cytotoxicity *in vitro* against the human leukemia cell line (HL-60), human lung adenocarcinoma epithelial cell line (A549) and human breast cancer cell line (MDA-MB-231) by the 3-(4,5-dimethylthiazol-2-yl)-2,5-diphenyltetrazolium bromide (MTT) assay, taking sorafenib and PAC-1 as references. The results expressed as IC_50_ values are summarized in Table 1. The IC_50_ values are the average of at least three independent experiments. As listed in Table 1, phenylhydrazones **1a**–**g** and **2a**–**k**, with the exception of **1c** and **1d**, exhibited moderate to excellent cytotoxicity towards the tested cell lines with IC_50_ values ranging from 0.22 to 6.0 μm. Generally, phenyl groups Ar^2^ substituted with chromenyl and imidazolidinyl moieties gave rise to two series of compounds **3a**–**e** and **4a**–**c** with a dramatic decrease or even a loss in antitumor potency, indicating that Ar^2^ was critical for the optimal activity and it is not tolerant ofr bulky and rigid heteroaromatic rings in this region.

**Table 1:** Structures and cytotoxicity of compounds **1a**–**g**, **2a**–**k**, **3a**–**e** and **4a**–**c** against HL-60, A549 and MDA-MB-231 cell lines.

Compd.	Ar^1^	Ar^2^	IC_50_ (μmol/L)		
			HL-60	A549	MDA-MB-231
**1a**	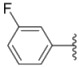	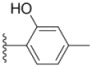	ND	0.64 ± 0.12	1.9 ± 0.16
**1b**	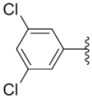	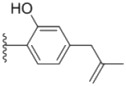	0.56 ± 0.04	0.78 ± 0.02	0.48 ± 0.02
**1c**	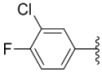	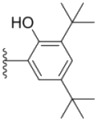	13.0 ± 0.37	0.48 ± 0.06	0.26 ± 0.01
**1d**	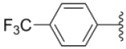	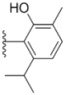	8.8 ± 0.31	5.1 ± 0.25	8.5 ± 0.44
**1e**	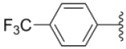	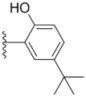	0.82 ± 0.08	1.6 ± 0.41	0.92 ± 0.24
**1f**	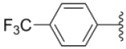	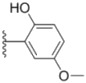	0.63 ± 0.17	1.3 ± 0.16	0.82 ± 0.05
**1g**	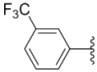	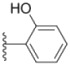	6.0 ± 0.09	0.50 ± 0.04	0.58 ± 0.03
**2a**	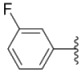	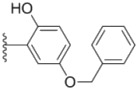	0.55 ± 0.09	1.6 ± 0.14	0.73 ± 0.06
**2b**	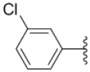	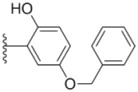	0.51 ± 0.01	1.2 ± 0.05	0.73 ± 0.02
**2c**	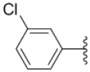	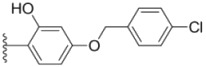	2.6 ± 0.11	0.59 ± 0.02	0.71 ± 0.01
**2d**	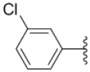	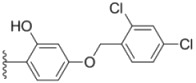	3.8 ± 0.13	1.7 ± 0.12	0.53 ± 0.02
**2e**	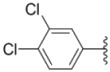	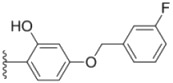	2.3 ± 0.11	0.49 ± 0.05	0.35 ± 0.02
**2f**	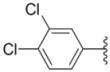	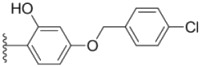	4.7 ± 0.19	2.8 ± 0.21	0.48 ± 0.05
**2g**	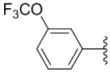	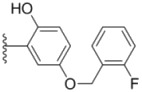	0.22 ± 0.01	0.34 ± 0.01	0.41 ± 0.3
**2h**	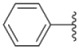	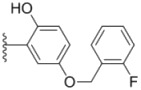	0.50 ± 0.004	1.8 ± 0.04	0.90 ± 0.006
**2i**	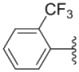	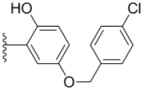	0.38 ± 0.01	0.54 ± 0.06	0.44 ± 0.04
**2j**	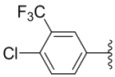	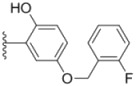	0.31 ± 0.14	0.96 ± 0.20	2.0 ± 0.12
**2k**	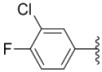	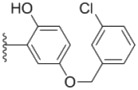	2.0 ± 0.11	2.3 ± 0.08	0.22 ± 0.04
**3a**	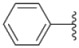	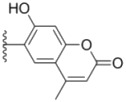	15.2 ± 0.22	17.0 ± 0.52	5.6 ± 0.36
**3b**	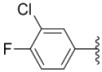	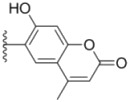	3.6 ± 0.12	>50	3.8 ± 0.28
**3c**	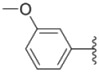	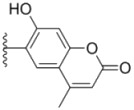	3.3 ± 0.25	6.4 ± 0.42	3.6 ± 0.28
**3d**	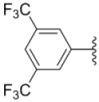	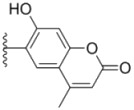	4.0 ± 0.33	1.7 ± 0.15	1.8 ± 0.07
**3e**	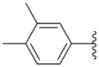	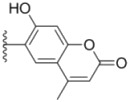	4.5 ± 0.13	19.0 ± 0.57	8.9 ± 0.41
**4a**	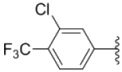	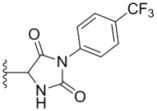	12.0 ± 0.32	37.2 ± 0.46	7.0 ± 0.18
**4b**	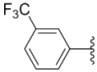	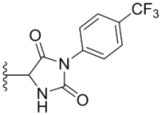	25.5 ± 0.29	3.4 ± 0.10	13.3 ± 0.32
**4c**	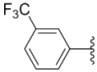	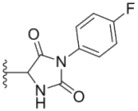	>50	7.8 ± 0.20	13.1 ± 0.37
Sorafenib			ND	1.3 ± 0.06	2.7 ± 0.11
PAC-1			4.5 ± 0.03	2.8 ± 0.10	2.0 ± 0.05

ND: not determined.

Compounds with alkyl groups on the phenyl ring Ar^2^ (compounds **1a**–**g**) exhibited moderate activity, however, the introduction of 4- or 5-benzyloxyl groups to the phenyl ring Ar^2^ resulted in a remarkable increase in the activity (**2a**–**c**, **2e**, **2g**–**k**). Moreover, compounds substituted with a 5-(2-fluorobenzyloxy) group (**2g** and **2j**) exhibited the most potent cytotoxicity, especially compound **2g** which displayed prominent activity with IC_50_ values of 0.22, 0.34 and 0.41 μM, respectively, which were 3.8- to 20.5-fold higher than those of sorafenib and PAC-1.

As for substituents on the ureido-linked phenyl Ar^1^, the introduction of electron-withdrawing substituents on Ar^1^ was beneficial to the improvement of cytotoxicity, and the trifluoromethoxyl group produced the best potency. A case in point is that compound **2g** with a trifluoromethoxyl group at the *meta*-position of Ar^1^ showed more potent cytotoxicity against all the three tested cell lines than compound **2h** with no substituent. Similarly, **3b** and **3d** with 3-trifluoromethyl and 3,5-di-trifluoromethyl groups on Ar^1^, respectively, displayed potency against two or three cell lines than **3a** with no substituent, while **3e** with an electron-donating 3,4-dimethyl group showed a decline in antitumor activity against the A549 and MDA-MB-231 cell lines.

## 3. Experimental

### 3.1. Chemistry

Melting points were obtained on a Büchi Melting Point B-540 apparatus (Büchi Labortechnik, Flawil, Switzerland) and were uncorrected. Mass spectra (MS) was taken in ESI mode on Agilent 1100 LC-MS (Agilent, Palo Alto, CA, USA). Proton (^1^H) nuclear magnetic resonance spectroscopy was performed using a Bruker ARX-300 300 MHz spectrometers (Bruker Bioscience, Billerica, MA, USA) with TMS as an internal standard. Unless otherwise noted, all the materials were obtained from commercial available sources and were used without further purification.

### 3.2. 4-Isocyanatobenzonitrile ***(5)***

4-Aminobezonitrile (100 g, 0.847 mol) was treated with excess hydrogen chloride-ethanol and the resulting solution was evaporated to dryness. The hydrochloride salt obtained was then dissolved in dioxane (200 mL) and added dropwise to a solution of triphosgene (125 g, 0.423 mol) in dioxane (200 mL). The reaction mixture was heated to 80 °C and stirred for 24 h. The resulting mixture was concentrated *in vacuo* and distilled under reduced pressure to give **5** (103 g, 84.4%) as colorless oil. B.p.: 130–131 °C (15 mmHg). 

### 3.3. General Procedure for Preparation of 1-(4-Cyanophenyl)-3-substituted Phenylureas ***6a–o***

To a solution of **5** (16 g, 0.111 mol) in tetrahydrofuran (100 mL) was slowly added the corresponding substituted aniline (0.111 mol). The resulted mixture was stirred at room temperature and the reaction was monitored by TLC. The reaction mixture was concentrated *in vacuo*, and the precipitated product was filtered and dried to obtain **6a**–**o**.

*1-(4-Cyanophenyl)-3-(3-fluorophenyl)urea* (**6a**): Yield: 87.2%; ESI-MS *m/z*: 256.1 [M+H]^+^. *1-(4-Cyanophenyl)-3-(3,5-dichlorophenyl)urea* (**6b**): Yield: 78.5%; ESI-MS *m/z*: 306.0 [M+H]^+^. *1-(4-Cyanophenyl-3-(3-chloro-4-fluorophenyl)urea* (**6c**): Yield: 84.7%; ESI-MS *m/z*: 290.0 [M+H]^+^. *1-(4-Cyanophenyl)-3-(4-(trifluoromethyl)phenyl)urea* (**6d**): Yield: 80.5%; ESI-MS *m/z*: 306.1 [M+H]^+^. *1-(4-Cyanophenyl)-3-(3-(trifluoromethyl)phenyl)urea* (**6e**): Yield: 80.0%; ESI-MS *m/z*: 306.1 [M+H]^+^. *1-(4-Cyanophenyl)-3-(3-chlorophenyl)urea* (**6f**): Yield: 88.5%; ESI-MS *m/z*: 272.0 [M+H]^+^. *1-(4-Cyanophenyl)-3-(3,4-dichlorophenyl)urea* (**6g**): Yield: 81.9%; ESI-MS *m/z*: 306.0 [M+H]^+^. *1-(4-Cyanophenyl)-3-(3-(trifluoromethoxy)phenyl)urea* (**6h**): Yield: 84.8%; ESI-MS *m/z*: 322.1 [M+H]^+^. *1-(4-Cyanophenyl)-3-phenylurea* (**6i**): Yield: 79.8%; ESI-MS *m/z*: 238.1 [M+H]^+^. *1-(4-Cyanophenyl)-3-(2-(trifluoromethyl)phenyl)urea* (**6j**): Yield: 83.4%; ESI-MS *m/z*: 306.1 [M+H]^+^. *1-(4-Cyanophenyl)-3-(4-chloro-3-(trifluoromethyl)phenyl)urea* (**6k**): Yield: 80.2%; ESI-MS *m/z*: 340.0 [M+H]^+^. *1-(4-Cyanophenyl)-3-(3-methoxyphenyl)urea* (**6l**): Yield: 83.0%; ESI-MS *m/z*: 268.1 [M+H]^+^. *-(4-Cyanophenyl)-3-(3,5-di(trifluoromethyl)phenyl)urea* (**6m**): Yield: 82.8%; ESI-MS *m/z*: 373.4 [M+H]^+^. *1-(4-Cyanophenyl)-3-(3,4-dimethylphenyl)urea* (**6n**): Yield: 82.4%; ESI-MS *m/z*: 266.1 [M+H]^+^. *1-(4-Cyanophenyl)-3-(3-chloro-4-(trifluoromethyl)phenyl)urea* (**6o**): Yield: 83.5%; ESI-MS *m/z*: 340.0 [M+H]^+^.

### 3.4. General Procedure for Preparation of 4-(3-Substituted Phenylureido)benzothioamides ***7a–o***

To a solution of benzonitrile **6a**–**o** (0.086 mol) in *N*,*N*-dimethylformamide (250 mL) was added magnesium chloride (22 g, 0.109 mol) and sodium hydrogensulfide (12.2 g, 0.218 mol ). The reaction mixture was stirred at room temperature overnight and then was added to 1 L water, acidified to pH 4 with dilute hydrochloric acid and filtered. The collected solid was washed with water until the filtrate became neutral and dried to obtain **7a**–**o**.

*4-(3-(3-Fuorophenyl)ureido)benzothioamide* (**7a**): Yield: 58.7%; ESI-MS *m/z*: 290.1 [M+H]^+^. *4-(3-(3,5-Dichlorophenyl)ureido)benzothioamide* (**7b**): Yield: 58.8%; ESI-MS *m/z*: 340.0 [M+H]^+^.* 4-(3-(3-Chloro-4-fluorophenyl)ureido)benzothioamide* (**7c**): Yield: 62.5%; ESI-MS *m/z*:324.0 [M+H]^+^. *4-(3-(4-(Trifluoromethyl)phenyl)ureido)benzothioamide* (**7d**): Yield: 60.7%; ESI-MS *m/z*: 340.1 [M+H]^+^. *4-(3-(3-(Trifluoromethyl)phenyl)ureido)benzothioamide* (**7e**): Yield: 66.4%; ESI-MS *m/z*: 340.1 [M+H]^+^. *4-(3-(3-Chlorophenyl)ureido)benzothioamide* (**7f**): Yield: 59.4%; ESI-MS *m/z*: 306.0 [M+H]^+^. *4-(3-(3,4-Dichlorophenyl)ureido)benzothioamide* (**7g**): Yield: 67.0%; ESI-MS *m/z*: 340.0 [M+H]^+^.* 4-(3-(3-(Trifluoromethoxy)phenyl)ureido)benzothioamide* (**7h**): Yield: 59.2%; ESI-MS *m/z*: 356.1 [M+H]^+^. *4-(3-Phenylureido)benzothioamide* (**7i**): Yield: 66.4%; ESI-MS *m/z*: 272.2 [M+H]^+^. *4-(3-(2-(Trifluoromethyl)phenyl)ureido)benzothioamide* (**7j**): Yield: 67.2%; ESI-MS *m/z*: 340.1 [M+H]^+^. *4-(3-(4-Chloro-3-(trifluoromethyl)phenyl)ureido)benzothioamide* (**7k**): Yield: 61.1%; ESI-MS *m/z*: 374.2 [M+H]^+^. *4-(3-(3-Methoxyphenyl)ureido)benzothioamide* (**7l**): Yield: 58.5%; ESI-MS *m/z*:302.1 [M+H]^+^. *4-(3-(3,5-Di(trifluoromethyl)phenyl)ureido)benzothioamide* (**7m**): Yield: 62.7%; ESI-MS *m/z*: 408.1 [M+H]^+^. *4-(3-(3,4-Dimethylphenyl)ureido)benzothioamide* (**7n**): Yield: 66.5%; ESI-MS *m/z*:300.3 [M+H]^+^. *4-(3-(3-Chloro-4-(trifluoromethyl)phenyl)ureido)benzothioamide* (**7o**): Yield: 63.1%; ESI-MS *m/z*: 374.2[M+H]^+^.

### 3.5. General Procedure for Preparation of 1-(4-(4-Chloromethylthiazol-2-yl)phenyl)-3-substituted Phenylureas ***8a–o***

Arylthioamides **7a**–**o** (0.083 mol) were dissolved in tetrahydrofuran (300 mL) and heated to 50 °C. To the stirred solution was added 1,3-dichloroacetone (10.5 g, 0.083 mol). The reaction mixture was stirred for 7 h. The resulting mixture was evaporated *in vacuo* to remove most of the solvent, cooled and filtered off. The residue was suspended in 1 L water and the suspension was stirred and alkalinized to pH 8 with saturated potassium carbonate solution. The precipitates was filtered, washed with water and dried to obtain **8a**–**o**.

*1-(4-(4-(Chloromethyl)thiazol-2-yl)phenyl)-3-(3-fluorophenyl)urea* (**8a**): Yield: 50.3%; ESI-MS *m/z*: 362.1 [M+H]^+^. *1-(4-(4-(Chloromethyl)thiazol-2-yl)phenyl)-3-(3,5-dichlorophenyl)urea* (**8b**): Yield: 58.1%; ESI-MS *m/z*: 412.0 [M+H]^+^. *1-(4-(4-(Chloromethyl)thiazol-2-yl)phenyl)-3-(3-chloro-4-fluorophenyl)urea* (**8c**): Yield: 55.3%; ESI-MS *m/z*: 396.0 [M+H]^+^. *1-(4-(4-(Chloromethyl)thiazol-2-yl)phenyl)-3-(4-(trifluoromethyl)phenyl)urea* (**8d**): Yield: 53.5%; ESI-MS *m/z*: 412.1 [M+H]^+^. *1-(4-(4-(Chloromethyl)thiazol-2-yl)phenyl)-3-(3-(trifluoromethyl)phenyl)urea* (**8e**): Yield: 50.9%; ESI-MS *m/z*: 412.1 [M+H]^+^.* 1-(4-(4-(Chloromethyl)thiazol-2-yl)phenyl)-3-(3-chlorophenyl)urea* (**8f**): Yield: 51.2%; ESI-MS *m/z*: 378.0 [M+H]^+^. *1-(4-(4-(Chloromethyl)thiazol-2-yl)phenyl)-3-(3,4-dichlorophenyl)urea* (**8g**): Yield: 57.5%; ESI-MS *m/z*: 412.0 [M+H]^+^.* 1-(4-(4-(Chloromethyl)thiazol-2-yl)phenyl)-3-(3-(trifluoromethoxy)phenyl)urea* (**8h**): Yield: 57.6%; ESI-MS *m/z*: 428.1 [M+H]^+^. *1-(4-(4-(Chloromethyl)thiazol-2-yl)phenyl)-3-phenylurea* (**8i**): Yield: 54.3%; ESI-MS *m/z*: 344.1 [M+H]^+^.* 1-(4-(4-(Chloromethyl)thiazol-2-yl)phenyl)-3-(2-(trifluoromethyl)phenyl)urea* (**8j**): Yield: 52.7%; ESI-MS *m/z*: 412.1 [M+H]^+^. *1-(4-(4-(Chloromethyl)thiazol-2-yl)phenyl)-3-(4-chloro-3-(trifluoromethyl)phenyl)urea* (**8k**): Yield: 55.6%; ESI-MS *m/z*: 446.0 [M+H]^+^. *1-(4-(4-(Chloromethyl)thiazol-2-yl)phenyl)-3-(3-methoxyphenyl)urea* (**8l**): Yield: 57.1%; ESI-MS *m/z*: 374.2 [M+H]^+^. *1-(4-(4-(Chloromethyl)thiazol-2-yl)phenyl)-3-(3,5-di(trifluoromethyl)phenyl)urea* (**8m**): Yield: 52.3%; ESI-MS *m/z*: 480.0 [M+H]^+^. *1-(4-(4-(Chloromethyl)thiazol-2-yl)phenyl)-3-(3,4-dimethylphenyl)urea* (**8n**): Yield: 50.4%; ESI-MS *m/z*: 372.2 [M+H]^+^. *1-(4-(4-(Chloromethyl)thiazol-2-yl)phenyl)-3-(3-chloro-4-(trifluoromethyl)phenyl)urea* (**8o**): Yield: 54.2%; ESI-MS *m/z*: 446.0 [M+H]^+^.

### 3.6. General Procedure for Preparation of 1-Substituted Phenyl-3-(4-(4-(piperazin-1-ylmethyl)thiazol-2-yl)phenyl)ureas ***9a–o***

To a solution of piperazine (64 g, 0.748 mol) in ethanol was added urea **8a**–**o** in portions. The reaction mixture was stirred at room temperature for 2 h. The resulting mixture was evaporated *in vacuo* to remove most of the solvent and poured into 1.5 L water. The white precipitates was filtered, washed with water and dried to obtain **9a**–**o**.

*1-(3-Fluorophenyl)-3-(4-(4-(piperazin-1-ylmethyl)thiazol-2-yl)phenyl)urea* (**9a**): Yield: 78.1%; ESI-MS *m/z*: 412.3 [M+H]^+^. *1-(3,5-Dichlorophenyl)-3-(4-(4-(piperazin-1-ylmethyl)thiazol-2-yl)phenyl)urea* (**9b**): Yield: 79.6%; ESI-MS *m/z*: 462.1 [M+H]^+^. *1-(3-Chloro-4-fluorophenyl)-3-(4-(4-(piperazin-1-ylmethyl)thiazol-2-yl)phenyl)urea* (**9c**): Yield: 83.5%; ESI-MS *m/z*: 446.2 [M+H]^+^.* 1-(4-(Trifluoromethyl)phenyl)-3-(4-(4-(piperazin-1-ylmethyl)thiazol-2-yl)phenyl)urea* (**9d**): Yield: 81.0%; ESI-MS *m/z*: 463.2 [M+H]^+^. *1-(3-(Trifluoromethyl)phenyl)-3-(4-(4-(piperazin-1-ylmethyl)thiazol-2-yl)phenyl)urea* (**9e**): Yield: 83.4%; ESI-MS *m/z*: 463.2 [M+H]^+^. *1-(3-Chlorophenyl)-3-(4-(4-(piperazin-1-ylmethyl)thiazol-2-yl)phenyl)urea* (**9f**): Yield: 77.7%; ESI-MS *m/z*: 428.2 [M+H]^+^. *1-(3,4-Dichlorophenyl)-3-(4-(4-(piperazin-1-ylmethyl)thiazol-2-yl)phenyl)urea* (**9g**): Yield: 78.5%; ESI-MS *m/z*: 462.1 [M+H]^+^. *1-(3-(Trifluoromethoxy)phenyl)-3-(4-(4-(piperazin-1-ylmethyl)thiazol-2-yl)phenyl)urea* (**9h**): Yield: 80.9%; ESI-MS *m/z*: 478.3 [M+H]^+^. *1-phenyl-3-(4-(4-(piperazin-1-ylmethyl)thiazol-2-yl)phenyl)urea* (**9i**): Yield: 83.4%; ESI-MS *m/z*: 394.2 [M+H]^+^. *1-(2-(Trifluoromethyl)phenyl)-3-(4-(4-(piperazin-1-ylmethyl)thiazol-2-yl)phenyl)urea* (**9j**): Yield: 55.6%; ESI-MS *m/z*: 463.2 [M+H]^+^. *1-(4-Chloro-3-(trifluoromethyl)phenyl)-3-(4-(4-(piperazin-1-ylmethyl)thiazol-2-yl)phenyl)urea* (**9k**): Yield: 86.8%; ESI-MS *m/z*: 480.2 [M+H]^+^. *1-(3-Methoxyphenyl)-3-(4-(4-(piperazin-1-ylmethyl)thiazol-2-yl)phenyl)urea* (**9l**): Yield: 78.9%; ESI-MS *m/z*: 424.3 [M+H]^+^. *1-(3,5-Di(trifluoromethyl)phenyl)-3-(4-(4-(piperazin-1-ylmethyl)thiazol-2-yl)phenyl)urea* (**9m**): Yield: 79.6%; ESI-MS *m/z*: 530.2 [M+H]^+^. *1-3,4-Dimethylphenyl)phenyl)-3-(4-(4-(piperazin-1-ylmethyl)thiazol-2-yl)phenyl)urea* (**9n**): Yield: 75.0%; ESI-MS *m/z*: 422.2 [M+H]^+^. *1-(3-Chloro-4-(trifluoromethyl)phenyl)-3-(4-(4-(piperazin-1-ylmethyl)thiazol-2-yl)phenyl)urea* (**9o**): Yield: 79.4%; ESI-MS *m/z*: 480.2 [M+H]^+^.

### 3.7. General Procedure for Preparation of Ethyl 2-(4-((2-(4-(3-substituted phenylureido)phenyl)thiazol-4-yl)methyl)piperazin-1-yl)acetates ***10a–o***

To a solution of *N*-substituted piperazine **9a**–**o** (0.063 mol) in ethanol (300 mL) was added potassium carbonate (5.2 g, 0.038 mol), ethyl chloroacetate (7.7 g, 0.063 mol) and sodium iodide (cat.). The reaction mixture was heated to 50 °C and stirred for 2 h. The reaction mixture was concentrated *in vacuo* and cooled. The product precipitated was filtered off, washed with ethanol and water, and dried to obtain **10a**–**o**.

*Ethyl 2-(4-((2-(4-(3-(3-fluorophenyl)ureido)phenyl)thiazol-4-yl)methyl)piperazin-1-yl)acetate* (**10a**): Yield: 87.7%; ESI-MS *m/z*: 498.2 [M+H]^+^. *Ethyl 2-(4-((2-(4-(3-(3,5-dichlorophenyl)ureido)phenyl)thiazol-4-yl)methyl)piperazin-1-yl)acetate* (**10b**): Yield: 88.3%; ESI-MS *m/z*: 548.2 [M+H]^+^. *Ethyl 2-(4-((2-(4-(3-(3-chloro-4-fluorophenyl)ureido)phenyl)thiazol-4-yl)methyl)piperazin-1-yl)acetate* (**10c**): Yield: 90.4%; ESI-MS *m/z*:532.2 [M+H]^+^. *Ethyl 2-(4-((2-(4-(3-(4-(trifluoromethyl)phenyl)ureido)phenyl)thiazol-4-yl)methyl)piperazin-1-yl)acetate* (**10d**): Yield: 93.4%; ESI-MS *m/z*: 548.2 [M+H]^+^. *Ethyl 2-(4-((2-(4-(3-(3-(trifluoromethyl)phenyl)ureido)phenyl)thiazol-4-yl)methyl)piperazin-1-yl)acetate* (**10e**): Yield: 92.0%; ESI-MS *m/z*: 548.2 [M+H]^+^. *Ethyl 2-(4-((2-(4-(3-(3-chlorophenyl)ureido)phenyl)thiazol-4-yl)methyl)piperazin-1-yl)acetate* (**10f**): Yield: 89.1%; ESI-MS *m/z*: 514.2 [M+H]^+^. *Ethyl 2-(4-((2-(4-(3-(3,4-dichlorophenyl)ureido)phenyl)thiazol-4-yl)methyl)piperazin-1-yl)acetate* (**10g**): Yield: 87.0%; ESI-MS *m/z*: 548.1 [M+H]^+^. *Ethyl 2-(4-((2-(4-(3-(3-(trifluoromethoxy)phenyl)ureido)phenyl)thiazol-4-yl)methyl)piperazin-1-yl)acetate* (**10h**): Yield: 88.7%; ESI-MS *m/z*: 564.2 [M+H]^+^. *Ethyl 2-(4-((2-(4-(3-phenylureido)phenyl)thiazol-4-yl)methyl)piperazin-1-yl)acetate* (**10i**): Yield: 90.4%; ESI-MS *m/z*: 480.2 [M+H]^+^. *Ethyl 2-(4-((2-(4-(3-(2-(trifluoromethyl)phenyl)ureido)phenyl)thiazol-4-yl)methyl)piperazin-1-yl)acetate* (**10j**): Yield: 91.7%; ESI-MS *m/z*: 548.2 [M+H]^+^. *Ethyl 2-(4-((2-(4-(3-(4-chloro-3-(trifluoromethyl)phenyl)ureido)phenyl)thiazol-4-yl)methyl)piperazin-1-yl)acetate* (**10k**) Yield: 91.5%; ESI-MS *m/z*: 582.1 [M+H]^+^. *Ethyl 2-(4-((2-(4-(3-(3-methoxyphenyl)ureido)phenyl)thiazol-4-yl)methyl)piperazin-1-yl)acetate* (**10l**) Yield: 93.4%; ESI-MS *m/z*: 498.2 [M+H]^+^. *Ethyl 2-(4-((2-(4-(3-(3,5-di(trifluoromethyl)phenyl)ureido)phenyl)thiazol-4-yl)methyl)piperazin-1-yl)acetate* (**10m**): Yield: 95.0%; ESI-MS *m/z*: 616.2 [M+H]^+^. *Ethyl 2-(4-((2-(4-(3-(3,4-dimethylphenyl)ureido)phenyl)thiazol-4-yl)methyl)piperazin-1-yl)acetate* (**10n**) Yield: 88.8%; ESI-MS *m/z*: 508.3 [M+H]^+^. *Ethyl 2-(4-((2-(4-(3-(3-chloro-4-(trifluoromethyl)phenyl)ureido)phenyl)thiazol-4-yl)methyl)piperazin-1-yl)acetate* (**10o**): Yield: 88.4%; ESI-MS *m/z*: 582.1 [M+H]^+^.

### 3.8. General Procedure for Preparation of 1-Substituted phenyl-3-(4-(4-((4-(2-hydrazinyl-2-oxoethyl)piperazin-1-yl)methyl)thiazol-2-yl)phenyl)ureas ***11a–o***

To a solution of ester **10a**–**o** (0.044 mol) in ethanol (250 mL) was added 80% hydrazine hydrate (27.7 g, 0.443 mol). The reaction mixture was heated to 50 °C and stirred for 48 h. The reaction mixture was evaporated to remove most of the solvent. The residue was filtered off, washed with water, and dried to obtain **11a**–**o**.

*1-(3-Fluorophenyl)-3-(4-(4-((4-(2-hydrazinyl-2-oxoethyl)piperazin-1-yl)methyl)thiazol-2-yl)phenyl)urea* (**11a**): Yield: 85.1%; ESI-MS *m/z*: 484.2 [M+H]^+^. *1-(3,5-Dichlorophenyl)-3-(4-(4-((4-(2-hydrazinyl-2-oxoethyl)piperazin-1-yl)methyl)thiazol-2-yl)phenyl)urea* (**11b**): Yield: 83.7%; ESI-MS *m/z*: 534.2 [M+H]^+^. *1-(3-Chloro-4-fluorophenyl)-3-(4-(4-((4-(2-hydrazinyl-2-oxoethyl)piperazin-1-yl)methyl)thiazol-2-yl)phenyl)urea* (**11c**): Yield: 88.9%; ESI-MS *m/z*:518.1 [M+H]^+^. *1-(4-(Trifluoromethyl)phenyl)-3-(4-(4-((4-(2-hydrazinyl-2-oxoethyl)piperazin-1-yl)methyl)thiazol-2-yl)phenyl)urea* (**11d**): Yield: 85.3%; ESI-MS *m/z*: 534.1 [M+H]^+^. *1-(3-(Trifluoromethyl)phenyl)-3-(4-(4-((4-(2-hydrazinyl-2-oxoethyl)piperazin-1-yl)methyl)thiazol-2-yl)phenyl)urea* (**11e**): Yield: 86.7%; ESI-MS *m/z*: 534.1 [M+H]^+^. *1-(3-Chlorophenyl)-3-(4-(4-((4-(2-hydrazinyl-2-oxoethyl)piperazin-1-yl)methyl)thiazol-2-yl)phenyl)urea* (**11f**): Yield: 85.1%; ESI-MS *m/z*: 500.2 [M+H]^+^. *1-(3,4-Dichlorophenyl)-3-(4-(4-((4-(2-hydrazinyl-2-oxoethyl)piperazin-1-yl)methyl)thiazol-2-yl)phenyl)urea* (**11g**): Yield: 87.5%; ESI-MS *m/z*: 534.2 [M+H]^+^. *1-(3-(Trifluoromethoxy)phenyl)-3-(4-(4-((4-(2-hydrazinyl-2-oxoethyl)piperazin-1-yl)methyl)thiazol-2-yl)phenyl)urea* (**11h**): Yield: 85.4%; ESI-MS *m/z*: 550.3 [M+H]^+^. *1-phenyl-3-(4-(4-((4-(2-hydrazinyl-2-oxoethyl)piperazin-1-yl)methyl)thiazol-2-yl)phenyl)urea* (**11i**): Yield: 87.8%; ESI-MS *m/z*: 466.2 [M+H]^+^. *1-(2-(Trifluoromethyl)phenyl)-3-(4-(4-((4-(2-hydrazinyl-2-oxoethyl)piperazin-1-yl)methyl)thiazol-2-yl)phenyl)urea* (**11j**): Yield: 88.1%; ESI-MS *m/z*: 534.1 [M+H]^+^. *1-(4-Chloro-3-(trifluoromethyl)phenyl)-3-(4-(4-((4-(2-hydrazinyl-2-oxoethyl)piperazin-1-yl)methyl)thiazol-2-yl)phenyl)urea* (**11k**): Yield: 88.0%; ESI-MS *m/z*: 568.2 [M+H]^+^. *1-(3-Methoxyphenyl)-3-(4-(4-((4-(2-hydrazinyl-2-oxoethyl)piperazin-1-yl)methyl)thiazol-2-yl)phenyl)urea* (**11l**): Yield: 85.2%; ESI-MS *m/z*: 496.3 [M+H]^+^. *1-(3,5-Di(trifluoromethyl)phenyl)-3-(4-(4-((4-(2-hydrazinyl-2-oxoethyl)piperazin-1-yl)methyl)thiazol-2-yl)phenyl)urea* (**11m**): Yield: 84.7%; ESI-MS *m/z*: 602.2 [M+H]^+^. *1-(3,4-Dimethylphenyl)-3-(4-(4-((4-(2-hydrazinyl-2-oxoethyl)piperazin-1-yl)methyl)thiazol-2-yl)phenyl)urea* (**11n**): Yield: 88.2%; ESI-MS *m/z*: 494.3 [M+H]^+^. *1-(3-Chloro-4-(trifluoromethyl)phenyl)-3-(4-(4-((4-(2-hydrazinyl-2-oxoethyl)piperazin-1-yl)methyl)thiazol-2-yl)phenyl)urea* (**11o**): Yield: 87.34%; ESI-MS *m/z*: 568.2 [M+H]^+^.

### 3.9. General Procedure for Preparation of 5-Benzyloxy-2-hydroxybenzaldehydes ***12a–d***

To a solution of 2,5-dihydroxybenzaldehyde (50 g, 0.362 mol) in acetonitrile (500 mL) was added substituted benzyl chloride (0.471 mol), sodium hydrogen carbonate (35 g, 0.413 mol) and potassium iodide (cat.). The reaction mixture was heated to reflux and stirred for 30 h. The mixture was poured into water (500 mL). The precipitates were filtered off, dried, recrystallized from methanol to give **12a**–**d**.

*5-(Benzyloxy)-2-hydroxybenzaldehyde* (**12a**): Yield: 53.5%; ESI-MS *m/z*: 227.1 [M+H]^+^. *5-((2-Fluorobenzyl)oxy)-2-hydroxybenzaldehyde* (**12b**): Yield: 48.8%; ESI-MS *m/z*: 245.1 [M+H]^+^. *5-((4-Chlorobenzyl)oxy)-2-hydroxybenzaldehyde *(**12c**): Yield: 45.5%; ESI-MS *m/z*:261.0 [M+H]^+^. *5-((3-Chlorobenzyl)oxy)-2-hydroxybenzaldehyde* (**12d**): Yield: 46.9%; ESI-MS *m/z*: 261.0 [M+H]^+^.

### 3.10. General Procedure for Preparation of 4-Benzyloxy-2-hydroxybenzaldehydes ***13a–c***

To a solution of 2,4-dihydroxybenzaldehyde (50 g, 0.362 mol) in acetonitrile (500 mL) was added benzyl chloride (60 g, 0.471 mol), sodium hydrogencarbonate (35 g, 0.413 mol) and potassium iodide (cat.). The reaction mixture was heated to reflux and stirred for 30 h. The mixture was poured into water (500 mL). The precipitates were filtered off, dried, recrystallized from methanol to give **13a**–**c**.

*4-((4-Chlorobenzyl)oxy)-2-hydroxybenzaldehyde* (**13a**): Yield: 49.1%; ESI-MS *m/z*: 260.9 [M+H]^+^. *4-((2,4-Dichlorobenzyl)oxy)-2-hydroxybenzaldehyde* (**13b**): Yield: 50.4%; ESI-MS *m/z*: 295.0 [M+H]^+^. *4-((3-Fluorobenzyl)oxy)-2-hydroxybenzaldehyde *(**13c**): Yield: 49.7%; ESI-MS *m/z*: 245.1 [M+H]^+^.

### 3.11. 7-Hydroxy-4-methyl-2H-chromen-2-one ***(14)***

Sulfuric acid (500 mL) was cooled below 10 °C in an ice-salt bath, and to the cooled acid was slowly added a solution of *m*-dihydroxybenzene (55 g, 0.500 mol) in ethyl acetoacetate (65 g, 0.500 mol). The reaction mixture was stirred for 4 h. The resulting mixture was poured onto cracked ice and stirred. The precipitates were filtered off, washed with water and dried to afford **14** (67.0 g, 76.1%) as a white solid. ESI-MS *m/z: *175.0 [M+H]^+^.

### 3.12. 7-Hydroxy-4-methyl-2-oxo-2H-chromene-6-carbaldehyde ***(15)***

A suspension of urotropine (20 g, 0.143 mol) in glacial acetic acid (80 mL) was heated to 40 °C and stirred until a clear solution formed. To this solution was added **14** (5 g, 0.028 mol) in portions. The reaction mixture was stirred for 20 min and then heated to 115 °C and stirred for another 2 h. Finally the reaction mixture was cooled to 95 °C, 30% sulfuric acid (15 mL) was added and the reaction continued for 1.5 h. The resulting mixture was evaporated to dryness and the dry residue obtained was extracted with ether. The organic layer was then washed with saturated sodium hydrogencarbonate solution, dried over anhydrous sodium sulfate and evaporated to dryness to afford **15** (2.2 g, 38.0%) as a white solid. ESI-MS *m/z*: 205.0 [M+H]^+^.

### 3.13. General Procedure for Preparation of Substituted 4-Substituted Phenyl Isocyanates ***16a–b***

To a solution of triphosgene (40 g, 0.135 mol) in dioxane (100 mL) was added substituted aniline (0.270 mol) dropwise. The reaction mixture was heated to 80 °C and stirred for 24 h. The reaction mixture was evaporated *in vacuo* to remove the solvent. The residue was distilled under reduced pressure to give the desired phenyl isocyanates **16a**–**b**.

*1-Isocyanato-4-(trifluoromethyl)benzene* (**16a**): Yield: 70.6%; b.p.: 90–92 °C (20 mmHg). *1-Fluoro-4-isocyanatobenzene* (**16b**): Yield: 73.1%; b.p.: 75–77 °C (20 mmHg).

### 3.14. General Procedure for Preparation of Methyl 2-(3-(4-Substituted phenyl)ureido)acetates ***17a–b***

To a suspension of glycine methyl ester hydrochloride (16 g, 0.130 mol) in dichloromethane (150 mL) was added triethylamine (15.6 g, 0.154 mol) dropwise. The mixture was stirred at room temperature for 1 h and cooled below 0 °C in an ice bath. To the cold mixture was added isocyanate **16a**–**b** (0.118 mol) slowly. The reaction mixture was kept in an ice bath and stirred for 4 h, then another 1 h at room temperature. The reaction mixture was evaporated to dryness, poured into water (300 mL) and stirred. The precipitates were collected by filtration and dried to give **17a**–**b**.

*Methyl 2-(3-(4-(trifluoromethyl)phenyl)ureido)acetate* (**17a**): Yield: 80.6%; ESI-MS *m/z*: 277.1 [M+H]^+^. *Methyl 2-(3-(4-fluorophenyl)ureido)acetate* (**17b**): Yield: 77.8%; ESI-MS *m/z*: 227.1 [M+H]^+^.

### 3.15. General Procedure for Preparation of 3-(4-Substituted phenyl)imidazolidine-2,4-diones ***18a–b***

To a solution of ester **17a**–**b** (0.044 mol) in acetone (100 mL) was added concentrated hydrochloride (110 mL). The reaction mixture was stirred and refluxed for 4 h. The reaction mixture was evaporated to remove most of the solvent and cooled. The precipitates were filtered off, washed by water and dried to give **18a**–**b**.

*3-(4-(Trifluoromethyl)phenyl)imidazolidine-2,4-dione* (**18a**): Yield: 66.7%; ESI-MS *m/z*: 245.1 [M+H]^+^. *3-(4-Fluorophenyl)imidazolidine-2,4-dione* (**18b**): Yield: 61.2%; ESI-MS *m/z*: 195.1 [M+H]^+^.

### 3.16. General Procedure for Preparation of 5-((dimethylamino)methylene)-3-(4-substituted phenyl)imidazolidine-2,4-diones ***19a–b***

Imidazolindione **18a**–**b** (0.026 mol), *N,N*-dimethylformamide dimethylacetal (12 g, 0.103 mol) were added to acetonitrile (10 mL). The reaction mixture was stirred and refluxed for 4 h. The reaction mixture was cooled, and precipitates were filtered off, washed by acetonitrile in small portions, dried to give **19a**–**b**.

*5-((Dimethylamino)methylene)-3-(4-(trifluoromethyl)phenyl)imidazolidine-2,4-dione* (**19a**) Yield: 47.7%; ESI-MS *m/z*: 277.1 [M+H]^+^. *5-((Dimethylamino)methylene)-3-(4-fluorophenyl)imidazolidine-2,4-dione ***(19b**): Yield: 52.4%; ESI-MS *m/z*: 227.1 [M+H]^+^. 

### 3.17. General Procedure for the Preparation of Target Compounds ***1a–g***, ***2a–k***, and ***3a–e***

To a solution of acethydrazide **11a**–**o** (0.002 mol) in ethanol (10 mL) was added appropriate benzaldehyde or the prepared aromatic aldehyde **12a**–**d**, **13a**–**c** or **15**. The reaction mixture was stirred and refluxed for 2 h. The reaction mixture was cooled and precipitates were collected by filtration to obtain the crude product, which was then purified by flash column chromatography. The pure product was dissolved in chloroform. To the solution was added excess hydrogen chloride-ethanol and stirred for 1 h. Ether was added to the mixture above. The precipitates were filtered off and dried to afford **1a**–**g**, **2a**–**k**, and **3a**–**e** as dihydrochlorides.

*1-(3-Fluorophenyl)-3-(4-(4-((4-(2-(2-(2-hydroxy-4-methylbenzylidene)hydrazinyl)-2-oxoethyl)piperazin-1-yl)methyl)thiazol-2-yl)phenyl)urea* (**1a**). Yield: 80.1%; M.p.: 185–187 °C; ESI-MS *m/z*: 602.1 [M−2HCl+H]^+^; ^1^H-NMR (DMSO-*d_6_*) *δ* (ppm): 11.66 (s, 1H), 11.50 (s, 1H), 10.44 (s, 1H), 9.03 (s, 1H), 8.97 (s, 1H), 8.42 (s, 1H), 7.86 (d, 2H), 7.58 (d, 2H), 7.50 (d, 1H), 7.41 (s, 1H), 7.32 (dd, 1H), 7.15 (d, 1H), 7.04 (s, 1H), 6.98 (s, 1H), 6.80 (t, 1H), 3.66 (s, 2H), 3.34 (s, 1H), 3.32 (s, 2H), 3.12 (s, 1H), 2.54 (s, 6H), 2.22 (s, 3H). Anal. Calcd for C_31_H_32_FN_7_O_3_S (%):C, 61.88; H, 5.36; N, 16.30; Found (%): C, 61.81; H, 5.42; N, 16.28.

*1-(3,5-Dichlorophenyl)-3-(4-(4-((4-(2-(2-(2-hydroxy-3-(2-methylallyl)benzylidene)hydrazinyl)-2-oxoethyl)piperazin-1-yl)methyl)thiazol-2-yl)phenyl)urea* (**1b**). Yield: 76.1%; M.p.: 178–180 °C; ESI-MS *m/z*: 691.7 [M−2HCl+H]^+^; ^1^H-NMR (DMSO-*d_6_*) *δ* (ppm): 11.89 (s, 1H), 11.50 (s, 1H), 9.20 (s, 1H), 9.15 (s, 1H), 8.49 (s, 1H), 7.86 (m, 3H), 7.57 (m, 3H), 7.41 (s, 1H), 7.25 (d, 1H), 7.18–7.14 (m, 2H), 6.88 (t, 1H), 4.75 (s, 1H), 4.63 (s, 1H), 3.66–3.58 (m, 4H), 3.13 (s, 2H), 2.54 (s, 8H), 1.67 (s, 3H). Anal. Calcd for C_34_H_35_Cl_2_N_7_O_3_S (%): C, 58.96; H, 5.09; N, 14.16; Found (%): C, 58.94; H, 5.02; N, 14.22.

*1-(3-Chloro-4-fluorophenyl)-3-(4-(4-((4-(2-(2-(3,5-di-tert-butyl-2-hydroxybenzylidene)hydrazinyl)-2-oxoethyl)piperazin-1-yl)methyl)thiazol-2-yl)phenyl)urea* (**1c**). Yield: 82.7%; M.p.: 231–232 °C; ESI-MS *m/z*: 734.6 [M−2HCl+H]^+^; ^1^H-NMR (DMSO-*d_6_*) *δ* (ppm): 11.98 (s, 1H), 9.71 (s, 1H), 9.67 (s, 1H), 9.09 (s, 1H), 8.47 (s, 1H), 7.91 (d, 3H), 7.81 (d, 1H), 7.62 (d, 2H), 7.44 (d, 1H), 7.38–7.33 (m, 2H), 7.22 (s, 1H), 4.45 (s, 2H), 3.67 (brs, 10H), 1.41 (d, 9H), 1.28 (d, 9H). Anal. Calcd for C_38_H_45_ClFN_7_O_3_S (%):C, 62.15; H, 6.18; N, 13.35; Found (%): C, 62.17; H, 6.11; N, 13.28.

*1-(4-(4-((4-(2-(2-(2-Hydroxy-6-isopropyl-3-methylbenzylidene)hydrazinyl)-2-oxoethyl)piperazin-1-yl)methyl)thiazol-2-yl)phenyl)-3-(4-(trifluoromethyl)phenyl)urea* (**1d**). Yield: 77.4%; M.p.: 185–187 °C; ESI-MS *m/z*: 694.5 [M−2HCl+H]^+^. ^1^H-NMR (DMSO-*d_6_*) *δ *(ppm): 11.98 (s, 1H), 10.14 (s, 1H), 10.05 (s, 1H), 9.01 (s, 1H), 8.54 (s, 1H), 8.37 (s, 1H), 7.98–7.93 (m, 3H), 7.70–7.64 (m, 6H), 7.33 (t, 1H), 6.90 (dd, 1H), 4.67 (s, 2H), 4.56 (s, 2H), 3.60 (brs, 8H), 1.28 (d, 9H). Anal. Calcd for C_35_H_38_F_3_N_7_O_3_S (%):C, 60.59; H, 5.52; N, 14.13; Found (%): C, 60.61; H, 5.51; N, 14.18.

*1-(4-(4-((4-(2-(2-(5-(tert-Butyl)-2-hydroxybenzylidene)hydrazinyl)-2-oxoethyl)piperazin-1-yl)methyl)thiazol-2-yl)phenyl)-3-(4-(trifluoromethyl)phenyl)urea* (**1e**). Yield: 75.1%; M.p.: 215–218 °C; ESI-MS *m/z*: 694.4 [M−2HCl+H]^+^; ^1^H-NMR (DMSO-*d_6_*) *δ* (ppm): 12.51 (s, 1H), 11.70 (s, 1H), 10.00 (s, 1H), 9.95 (s, 1H), 8.90 (s, 1H), 7.89 (s, 3H), 7.75–7.62 (m, 7H), 7.14 (d, 1H), 6.75 (d, 1H), 4.27–4.09 (m, 2H), 3.35 (s, 1H), 3.17 (s, 4H), 3.07 (s, 2H), 2.87 (s, 4H), 2.13 (s, 3H), 1.21 (s, 6H). Anal. Calcd for C_35_H_38_F_3_N_7_O_3_S (%):C, 60.59; H, 5.52; N, 14.13; Found (%): C, 60.51; H, 5.48; N, 14.16.

*1-(4-(4-((4-(2-(2-(2-Hydroxy-5-methoxybenzylidene)hydrazinyl)-2-oxoethyl)piperazin-1-yl)methyl)thiazol-2-yl)phenyl)-3-(4-(trifluoromethyl)phenyl)urea* (**1f**). Yield: 70.7%; M.p.: 198–200 °C; ESI-MS *m/z*: 667.9 [M−2HCl+H]^+^; ^1^H-NMR (DMSO-*d_6_*) *δ* (ppm): 12.00 (s, 1H), 10.22 (s, 1H), 10.12 (s, 1H), 8.52 (s, 1H), 8.35 (s, 1H), 7.98–7.92 (m, 4H), 7.70–7.62 (m, 7H), 7.28 (s, 1H), 6.89 (s, 2H), 4.67 (s, 1H), 4.57 (s, 2H), 3.72 (s, 3H), 3.64 (brs, 9H). Anal. Calcd for C_32_H_33_F_3_N_7_O_4_S (%):C, 57.56; H, 4.83; N, 14.68; Found (%): C, 57.53; H, 4.85; N, 14.71.

*1-(4-(4-((4-(2-(2-(2-Hydroxybenzylidene)hydrazinyl)-2-oxoethyl)piperazin-1-yl)methyl)thiazol-2-yl)phenyl)-3-(3-(trifluoromethyl)phenyl)urea* (**1g**). Yield: 78.4%; M.p.: 205–207 °C; ESI-MS *m/z*: 638.1 [M−2HCl+H]^+^; ^1^H-NMR (DMSO-*d_6_*) *δ* (ppm): 11.94 (s, 1H), 10.14 (s, 1H), 9.84 (s, 1H), 9.74 (d, 1H), 8.51 (s, 1H), 8.36 (s, 1H), 8.02 (s, 1H), 7.93 (d, 2H), 7.89–7.88 (m, 1H), 7.75 (d, 1H), 7.66–7.50 (m, 4H), 7.34–7.24 (m, 2H), 6.94–6.84 (m, 2H), 4.54 (s, 1H), 4.45 (s, 1H), 4.03 (s, 2H), 3.51 (brs, 8H). Anal. Calcd for C_31_H_30_F_3_N_7_O_3_S (%):C, 58.39; H, 4.74; N, 15.38; Found (%): C, 58.36; H, 4.75; N, 15.36.

*1-(4-(4-((4-(2-(2-(5-(Benzyloxy)-2-hydroxybenzylidene)hydrazinyl)-2-oxoethyl)piperazin-1-yl)methyl)thiazol-2-yl)phenyl)-3-(3-fluorophenyl)urea* (**2a**). Yield: 74.9%; M.p.: 190–192 °C; ESI-MS *m/z*: 694.5 [M−2HCl+H]^+^; ^1^H-NMR (DMSO-*d_6_*) *δ* (ppm): 11.41 (s, 1H), 10.66 (s, 1H), 9.72 (s, 1H), 9.09 (s, 1H), 9.03 (s, 1H), 8.47 (s, 1H), 7.85 (d, 2H), 7.59 (d, 2H), 7.50 (d, 1H), 7.47–7.25 (m, 7H), 7.23–7.08 (m, 2H), 6.97 (dd, 1H), 6.85–6.77 (m, 2H), 5.04 (s, 2H), 3.64 (d, 2H), 3.31 (s, 4H), 2.52 (s, 6H). Anal. Calcd for C_37_H_36_FN_7_O_4_S (%):C, 64.05; H, 5.23; N, 14.13; Found (%): C, 64.02; H,5.24; N, 14.09.

*1-(4-(4-((4-(2-(2-(5-(Benzyloxy)-2-hydroxybenzylidene)hydrazinyl)-2-oxoethyl)piperazin-1-yl)methyl)thiazol-2-yl)phenyl)-3-(3-chlorophenyl)urea* (**2b**). Yield: 78.3%; M.p.: 188–190 °C; ESI-MS *m/z*: 709.9 [M−2HCl+H]^+^; ^1^H-NMR (DMSO-*d_6_*) *δ* (ppm): 11.45 (s, 1H), 10.67 (s, 1H), 9.53 (d, 2H), 8.48 (s, 1H), 8.17 (s, 1H), 7.85 (d, 2H), 7.72 (s, 1H), 7.59 (d, 2H), 7.45–7.30 (m, 9H), 7.16 (dd, 1H), 7.04–6.90 (m, 2H), 6.86–6.80 (m, 1H), 5.04 (s, 2H), 3.64 (d, 2H), 3.32 (s, 4H), 2.54 (s, 6H). Anal. Calcd for C_37_H_36_ClN_7_O_4_S (%):C, 62.57; H, 5.11; N, 13.80; Found (%): C, 62.55; H,5.14; N, 13.73.

*1-(4-(4-((4-(2-(2-(4-((4-Chlorobenzyl)oxy)-2-hydroxybenzylidene)hydrazinyl)-2-oxoethyl)piperazin-1-yl)methyl)thiazol-2-yl)phenyl)-3-(3-chlorophenyl)urea* (**2c**). Yield: 84.4%; M.p.: 218–220 °C; ESI-MS *m/z*: 744.1 [M−2HCl+H]^+^; ^1^H-NMR (DMSO-*d_6_*) *δ* (ppm): 11.80 (s, 1H), 9.76 (s, 1H), 9.72 (s, 1H), 9.67 (s, 1H), 8.41 (s, 1H), 8.26 (s, 1H), 7.93 (s, 1H), 7.90 (s, 2H), 7.71 (s, 1H), 7.63 (d, 2H), 7.46 (s, 4H), 7.31 (s, 1H), 7.30 (s, 1H), 7.02–7.01 (m, 1H), 6.59–6.54 (m, 2H), 5.11 (d, 2H), 4.51 (s, 1H), 4.45 (s, 2H), 3.58 (brs, 9H). Anal. Calcd for C_37_H_35_Cl_2_N_7_O_4_S (%):C, 59.68; H, 4.74; N, 13.17; Found (%): C, 59.64; H,4.70; N, 13.13.

*1-(3-Chlorophenyl)-3-(4-(4-((4-(2-(2-(4-((2,4-dichlorobenzyl)oxy)-2-hydroxybenzylidene)hydrazinyl)-2-oxoethyl)piperazin-1-yl)methyl)thiazol-2-yl)phenyl)urea* (**2d**). Yield: 75.2%; M.p.: 218–220 °C; ESI-MS *m/z*: 778.0 [M−2HCl+H]^+^; ^1^H-NMR (DMSO-*d_6_*) *δ* (ppm): 11.82 (s, 1H), 9.78 (s, 1H), 9.74 (s, 1H), 9.70 (s, 1H), 8.27 (s, 1H), 7.94 (s, 2H), 7.91 (s, 2H), 7.71–7.70 (m, 3H), 7.66–7.59 (m, 4H), 7.50–7.45 (m, 1H), 7.31 (s, 1H), 7.30 (s, 1H), 7.04–7.01 (m, 1H), 6.61–6.56 (m, 2H), 5.15 (d, 2H), 4.53 (s, 1H), 4.46 (s, 2H), 3.66 (brs, 9H). Anal. Calcd for C_37_H_34_Cl_3_N_7_O_4_S (%): C, 57.04; H, 4.40; N, 12.58; Found (%): C, 57.06; H,4.38; N, 12.51.

*1-(3,4-Dichlorophenyl)-3-(4-(4-((4-(2-(2-(4-((3-fluorobenzyl)oxy)-2-hydroxybenzylidene)hydrazinyl)-2-oxoethyl)piperazin-1-yl)methyl)thiazol-2-yl)phenyl)urea* (**2e**). Yield: 79.4%; M.p.: 209–210 °C; ESI-MS *m/z*: 761.8 [M−2HCl+H]^+^; ^1^H-NMR (DMSO-*d_6_*) *δ* (ppm): 11.79 (s, 1H), 9.81 (s, 1H), 9.77 (s, 1H), 9.74 (s, 1H), 9.70 (s, 1H), 8.26 (s, 1H), 7.93–7.88 (m, 4H), 7.62 (d, 3H), 7.53 (d, 1H), 7.46–7.41 (m, 1H), 7.35 (dd, 1H), 7.29–7.25 (m, 2H), 7.20–7.14 (m, 1H), 6.61–6.55 (m, 2H), 5.15 (s, 1H), 5.12 (s, 1H), 4.50 (s, 1H), 4.45 (s, 2H), 3.52 (brs, 9H). Anal. Calcd for C_37_H_34_Cl_2_FN_7_O_4_S (%):C, 58.27; H, 4.49; N, 12.86; Found (%): C, 58.26; H,4.47; N, 12.89.

*1-(4-(4-((4-(2-(2-(4-((4-Chlorobenzyl)oxy)-2-hydroxybenzylidene)hydrazinyl)-2-oxoethyl)piperazin-1-yl)methyl)thiazol-2-yl)phenyl)-3-(3,4-dichlorophenyl)urea (**2f**)*. Yield: 73.5%; M.p.: 227–228 °C; ESI-MS *m/z*: 777.7 [M−2HCl+H]^+^; ^1^H-NMR (DMSO-*d_6_*) *δ* (ppm): 11.78 (s, 1H), 9.79 (s, 1H), 9.75 (s, 1H), 9.72 (s, 1H), 9.69 (s, 1H), 8.41 (s, 1H), 8.25 (s, 1H), 7.93–7.88 (m, 4H), 7.62 (d, 2H), 7.53 (d, 2H), 7.46 (s, 4H), 7.35 (dd, 1H), 6.59–6.53 (m, 2H), 5.12 (s, 1H), 5.09 (s, 1H), 4.49 (s, 1H), 4.43 (s, 2H), 3.53 (s, 9H). Anal. Calcd for C_37_H_34_Cl_3_N_7_O_4_S (%):C, 57.04; H, 4.40; N, 12.58; Found (%): C, 57.06; H,4.47; N, 12.59.

*1-(4-(4-((4-(2-(2-(5-((2-Fluorobenzyl)oxy)-2-hydroxybenzylidene)hydrazinyl)-2-oxoethyl)piperazin-1-yl)methyl)thiazol-2-yl)phenyl)-3-(3-(trifluoromethoxy)phenyl)urea* (**2g**). Yield: 71.2%; M.p.: 165–168 °C; ESI-MS *m/z*: 778.5 [M−2HCl+H]^+^; ^1^H-NMR (DMSO-*d_6_*) *δ* (ppm): 11.42 (s, 1H), 10.71 (s, 1H), 9.75 (s, 1H), 9.09 (s, 1H), 9.08 (s, 1H), 8.48 (s, 1H), 8.17 (s, 1H), 7.86 (d, 2H), 7.71 (s, 1H), 7.59 (d, 2H), 7.54 (d, 1H), 7.44–7.39 (m, 3H), 7.33–7.14 (m, 3H), 7.01–6.95 (m, 2H), 6.85 (d, 1H), 5.08 (s, 2H), 3.66 (s, 2H), 3.47 (s, 1H), 3.10 (s, 1H), 2.54 (s, 8H). Anal. Calcd for C_38_H_35_F_4_N_7_O_5_S (%): C, 58.68; H, 4.54; N, 12.61; Found (%): C, 58.66; H,4.59; N, 12.58.

*1-(4-(4-((4-(2-(2-(5-((2-Fluorobenzyl)oxy)-2-hydroxybenzylidene)hydrazinyl)-2-oxoethyl)piperazin-1-yl)methyl)thiazol-2-yl)phenyl)-3-phenylurea* (**2h**). Yield: 78.4%; M.p.: 202–203 °C; ESI-MS *m/z*: 694.4 [M−2HCl+H]^+^; ^1^H-NMR (DMSO-*d_6_*) *δ* (ppm): 11.40 (s, 1H), 10.70 (s, 1H), 9.74 (s, 1H), 8.96 (s, 1H), 8.74 (s, 1H), 8.48 (s, 1H), 7.84 (d, 2H), 7.59–7.52 (m, 3H), 7.47 (d, 2H), 7.40 (s, 2H), 7.32–7.20 (m, 4H), 7.16 (d, 1H), 7.01–6.97 (m, 2H), 6.85 (d, 1H), 5.08 (s, 2H), 3.66 (s, 1H), 3.29 (s, 4H), 3.10 (s, 1H), 2.54 (s, 6H). Anal. Calcd for C_37_H_36_FN_7_O_4_S (%):C, 64.05; H, 5.23; N, 14.13; Found (%): C, 64.02; H, 5.20; N, 14.18.

*1-(4-(4-((4-(2-(2-(5-((4-Chlorobenzyl)oxy)-2-hydroxybenzylidene)hydrazinyl)-2-oxoethyl)piperazin-1-yl)methyl)thiazol-2-yl)phenyl)-3-(2-(trifluoromethyl)phenyl)urea* (**2i**). Yield: 72.6%; M.p.: 247–249 °C; ESI-MS *m/z*: 778.0 [M−2HCl+H]^+^; ^1^H-NMR (DMSO-*d_6_*) *δ* (ppm): 12.03 (s, 1H), 10.65 (s, 1H), 10.33–10.26 (m, 3H), 8.50 (s, 3H), 8.33 (s, 1H), 7.86 (s, 1H), 7.71 (s, 2H), 7.47 (s, 3H), 7.36–7.29 (m, 4H), 6.98 (dd, 1H), 6.89 (d, 1H), 5.06 (s, 2H), 4.67 (s, 2H), 4.52 (s, 2H), 3.92–3.32 (s, 8H). Anal. Calcd for C_38_H_35_ClF_3_N_7_O_4_S (%):C, 58.65; H, 4.53; N, 12.60; Found (%): C, 58.63; H, 4.55; N, 12.61.

*1-(4-Chloro-3-(Trifluoromethyl)phenyl)-3-(4-(4-((4-(2-(2-(5-((2-fluorobenzyl)oxy)-2-hydroxybenzylidene)hydrazinyl)-2-oxoethyl)piperazin-1-yl)methyl)thiazol-2-yl)phenyl)urea* (**2j**). Yield: 74.5%; M.p.: 165–167 °C; ESI-MS *m/z*: 796.5 [M−2HCl+H]^+^; ^1^H-NMR (DMSO-*d_6_*) *δ* (ppm): 11.42 (s, 1H), 10.71 (s, 1H), 9.75 (s, 1H), 9.61 (s, 1H), 9.47 (s, 1H), 8.49 (s, 1H), 8.13 (s, 1H), 7.83 (d, 2H), 7.66–7.52 (m, 6H), 7.40 (s, 2H), 7.23–7.17 (m, 3H), 6.99 (d, 1H), 6.85 (d, 1H), 5.08 (s, 2H), 3.66 (s, 1H), 3.64 (s, 1H), 3.49 (s, 1H), 3.31 (s, 1H), 3.11 (s, 2H), 2.54 (s, 6H). Anal. Calcd for C_38_H_34_ClF_4_N_7_O_4_S (%):C, 57.32; H, 4.30; N, 12.31; Found (%): C, 57.33; H, 4.35; N, 12.25.

*1-(3-Chloro-4-fluorophenyl)-3-(4-(4-((4-(2-(2-(5-((3-chlorobenzyl)oxy)-2-hydroxybenzylidene)hydrazinyl)-2-oxoethyl)piperazin-1-yl)methyl)thiazol-2-yl)phenyl)urea* (**2k**). Yield: 73.3%; M.p.: 194–195 °C; ESI-MS *m/z*: 762.2 [M−2HCl+H]^+^; ^1^H-NMR (DMSO-*d_6_*) *δ* (ppm): 11.42 (s, 1H), 10.67 (s, 1H), 9.75 (s, 1H), 9.09 (s, 1H), 8.98 (s, 1H), 8.47 (s, 1H), 7.86–7.80 (m, 4H), 7.58 (d, 2H), 7.51 (s, 1H), 7.41 (s, 4H), 7.34 (d, 2H), 7.15 (d, 1H), 7.00–6.96 (m, 1H), 6.84 (d, 1H), 5.07 (s, 2H), 3.66 (s, 2H), 3.32 (s, 2H), 3.10 (s, 2H), 2.54 (s, 6H). Anal. Calcd for C_37_H_34_Cl_2_FN_7_O_4_S (%):C, 58.27; H, 4.49; N, 12.86; Found (%): C, 58.25; H, 4.45; N, 12.92.

*1-(4-(4-((4-(2-(2-((7-Hydroxy-4-methyl-2-oxo-2H-chromen-6-yl)methylene)hydrazinyl)-2-oxoethyl)piperazin-1-yl)methyl)thiazol-2-yl)phenyl)-3-phenylurea* (**3a**). Yield: 75.2%; M.p.: 243–245 °C; ESI-MS *m/z*: 652.1 [M−2HCl+H]^+^; ^1^H-NMR (DMSO-*d_6_*) *δ* (ppm): 12.19 (s, 1H), 9.46 (d, 1H), 9.18 (d, 1H), 9.00 (s, 1H), 8.61 (s, 1H), 7.91 (d, 3H), 7.71 (d, 1H), 7.62 (d, 2H), 7.00–6.95 (d, 2H), 7.29 (t, 2H), 7.00–6.95 (m, 2H), 6.26 (s, 1H), 4.47 (s, 2H), 3.47 (d, 10H), 2.41 (s, 3H). Anal. Calcd for C_34_H_33_N_7_O_5_S (%):C, 62.66; H, 5.10; N, 15.04; Found (%): C, 62.61; H, 5.13; N, 15.02.

*1-(4-(4-((4-(2-(2-((7-Hydroxy-4-methyl-2-oxo-2H-chromen-6-yl)methylene)hydrazinyl)-2-oxoethyl)piperazin-1-yl)methyl)thiazol-2-yl)phenyl)-3-(3-(trifluoromethyl)phenyl)urea* (**3b**). Yield: 71.7%; M.p.: 242–243 °C; ESI-MS *m/z*: 720.1 [M−2HCl+H]^+^; ^1^H-NMR (DMSO-*d_6_*) *δ* (ppm): 12.53 (s, 1H), 12.16 (s, 1H), 10.99 (s, 1H), 9.96 (s, 1H), 9.87 (s, 1H), 8.98 (s, 1H), 8.61 (s, 1H), 8.01 (s, 1H), 7.93 (d, 3H), 7.72 (t, 1H), 7.68–7.57 (m, 3H), 7.52 (t, 1H), 7.32 (d, 1H), 6.98–6.95 (m, 1H), 6.26 (s, 1H), 4.52 (s, 2H), 3.80 (s, 2H), 3.59 (brs, 8H), 2.41 (s, 3H). Anal. Calcd for C_35_H_32_F_3_N_7_O_5_S (%):C, 58.41; H, 4.48; N, 13.62; Found (%): C, 58.43; H, 4.43; N, 13.68.

*1-(4-(4-((4-(2-(2-((7-Hydroxy-4-methyl-2-oxo-2H-chromen-6-yl)methylene)hydrazinyl)-2-oxoethyl)piperazin-1-yl)methyl)thiazol-2-yl)phenyl)-3-(3-methoxyphenyl)urea* (**3c**). Yield: 75.3%; M.p.: 236–238 °C; ESI-MS *m/z*: 682.3 [M−2HCl+H]^+^; ^1^H-NMR (DMSO-*d_6_*) *δ* (ppm): 12.60 (s, 1H), 12.19 (s, 1H), 9.40 (s, 1H), 9.00 (s, 2H), 8.61 (s, 1H), 7.89 (s, 3H), 7.74 (d, 1H), 7.61 (d, 2H), 7.37 (d, *2H*), 6.97 (d, 1H), 6.88 (d, 2H), 6.26 (s, 1H), 4.47 (s, 2H), 3.72 (s, 3H), 3.61 (s, 10H), 2.41 (s, 3H). Anal. Calcd for C_35_H_35_N_7_O_6_S (%):C, 61.66; H, 5.17; N, 14.38; Found (%): C, 61.13; H, 5.14; N, 14.35.

*1-(3,5-bis(Trifluoromethyl)phenyl)-3-(4-(4-((4-(2-(2-((7-hydroxy-4-methyl-2-oxo-2H-chromen-6-yl)methylene)hydrazinyl)-2-oxoethyl)piperazin-1-yl)methyl)thiazol-2-yl)phenyl)urea* (**3d**). Yield: 70.1%; M.p.: 225–226 °C; ESI-MS *m/z*: 788.3 [M−2HCl+H]^+^; ^1^H-NMR (DMSO-*d_6_*) *δ* (ppm): 12.68 (s, 1H), 10.53 (d, 1H), 10.01 (d, 1H), 8.98 (s, 1H), 8.13 (s, 2H), 8.00–7.89 (m, 4H), 7.73 (t, 1H), 7.66 (d, 3H), 7.06–6.88 (m, 1H), 6.26 (d, 1H), 4.55 (s, 4H), 4.11–3.56 (m, 8H), 2.41 (s, 3H). Anal. Calcd for C_36_H_31_F_6_N_7_O_5_S (%):C, 54.89; H, 3.97; N, 12.45; Found (%): C, 54.88; H, 3.94; N, 12.40.

*1-(3,4-Dimethylphenyl)-3-(4-(4-((4-(2-(2-((7-hydroxy-4-methyl-2-oxo-2H-chromen-6-yl)methylene)hydrazinyl)-2-oxoethyl)piperazin-1-yl)methyl)thiazol-2-yl)phenyl)urea* (**3e**). Yield: 72.2%; M.p.: 246–248 °C; ESI-MS *m/z*: 666.3 [M−2HCl+H]^+^; ^1^H-NMR (DMSO-*d_6_*) *δ* (ppm): 12.56 (s, 1H), 12.16 (s, 1H), 11.00 (s, 1H), 9.71 (d, 1H), 9.26 (d, 1H), 8.98 (s, 1H), 7.93–7.88 (m, 3H), 7.75–7.70 (m, 1H), 7.61 (dd, 2H), 7.24–7.19 (m, 2H), 7.04–6.95 (m, 2H), 6.27–6.25 (m, 1H), 4.52 (s, 2H), 3.81 (s, 2H), 3.60 (brs, 8H), 2.41 (s, 3H), 2.19 (s, 3H), 2.16 (s, 3H). Anal. Calcd for C_36_H_37_N_7_O_5_S (%):C, 63.61; H, 5.49; N, 14.42; Found (%): C, 63.58; H, 5.54; N, 14.40.

### 3.18. General Procedure for Preparation of Target Compounds ***4a–c***

Acethydrazide **11e** or **11o** (0.002 mol) was dissolved in 1M hydrochloride (5 mL) to obtain the hydrochloride form. The resulting hydrochloride salt was then added to a solution of imidazolindione **19a**–**b** (0.002 mol) in ethanol (10 mL). The reaction mixture was stirred at room temperature for 4 h. The precipitates were filtered off and dried to obtain **4a**–**c** as dihydrochlorides.

*1-(3-Chloro-4-(trifluoromethyl)phenyl)-3-(4-(4-((4-(2-(2-((2,5-dioxo-1-(4-(trifluoromethyl)phenyl)imidazolidin-4-yl)methylene)hydrazinyl)-2-oxoethyl)piperazin-1-yl)methyl)thiazol-2-yl)phenyl)urea* (**4a**). Yield: 58.4%; M.p.: 239–241 °C; ESI-MS *m/z*: 822.0 [M−2HCl+H]^+^; ^1^H-NMR (DMSO-*d_6_*) *δ* (ppm): 10.63 (s, 1H), 9.98 (s, 2H), 9.77 (s, 1H), 8.72 (d, 1H), 8.12 (s, 1H), 7.94 (s, 1H), 7.92 (d, *J* = 3.1 Hz, 2H), 7.84 (d, 2H), 7.67 (d, 2H), 7.65–7.62 (t, 4H), 6.73 (d, 1H), 4.49 (s, 2H), 3.73 (s, 2H), 3.38 (brs, 8H). Anal. Calcd for C_35_H_30_ClF_6_N_9_O_4_S (%):C, 51.13; H, 3.68; N, 15.33; Found (%): C, 51.18; H, 3.64; N, 15.40.

*1-(4-(4-((4-(2-(2-((2,5-dioxo-1-(4-(Trifluoromethyl)phenyl)imidazolidin-4-yl)methylene)hydrazinyl)-2-oxoethyl)piperazin-1-yl)methyl)thiazol-2-yl)phenyl)-3-(3-(trifluoromethyl)phenyl)urea* (**4b**). Yield: 57.8%; M.p.: 205–207 °C; ESI-MS *m/z*: 788.0 [M−2HCl+H]^+^; ^1^H-NMR (DMSO-*d_6_*) *δ* (ppm): 10.32 (s, 1H), 9.61 (d, 3H), 8.02 (s, 1H), 7.89–7.83 (m, 5H), 7.70–7.50 (m, 9H), 7.32 (d, 1H), 4.43 (s, 2H), 3.32 (s, 10H). Anal. Calcd for C_35_H_31_F_6_N_9_O_4_S (%):C, 53.37; H, 3.97; N, 16.00; Found (%): C, 53.38; H, 3.94; N, 15.92.

*1-(4-(4-((4-(2-(2-((1-(4-Fluorophenyl)-2,5-dioxoimidazolidin-4-yl)methylene)hydrazinyl)-2-oxoethyl)piperazin-1-yl)methyl)thiazol-2-yl)phenyl)-3-(3-(trifluoromethyl)phenyl)urea* (**4c**). Yield: 55.2%; M.p.: 200–201 °C; ESI-MS *m/z*: 738.2 [M−2HCl+H]^+^; ^1^H-NMR (DMSO-*d_6_*) *δ* (ppm): 11.56 (s, 1H), 10.22 (s, 1H), 9.60–9.35 (m, 3H), 8.31 (d, 1H), 8.03 (s, 1H), 7.90 (d, 2H), 7.65–7.60 (m, 3H), 7.55–7.50 (m, 1H), 7.45–7.26 (m, 5H), 6.65 (d, 1H), 4.23 (s, 2H), 3.75 (s, 1H), 3.52–2.55 (m, 9H). Anal. Calcd for C_34_H_31_F_4_N_9_O_4_S (%):C, 55.35; H, 4.24; N, 17.09; Found (%): C, 55.38; H, 4.20; N, 17.12.

### 3.19. Evaluation of the Biological Activity

The cytotoxicity of target compounds **1a**–**g**, **2a**–**k**, **3a**–**e** and **4a**–**c** were evaluated against the A549, MDA-MB-231 and HL-60 cell lines by MTT method *in vitro*, with sorafenib and PAC-1 as the positive controls. The cancer cell lines were cultured in minimum essential medium (MEM) supplement with 10% fetal bovine serum (FBS). Approximately 4 × 10^3^cells, suspend in MEM medium, were plated onto each well of a 96-well plate and incubated in 5% CO_2_ at 37 °C for 24 h. The tested compounds at indicated final concentrations were added to the culture medium and the cell cultures were continued for 72 h. Fresh MTT was added to each well at a terminal concentration of 5 μg/mL and incubated with cells at 37 °C for 4 h. The formazan crystals were dissolved in 100 μL DMSO per well, and the absorbency at 492 nm (for absorbance of MTT formazan) and 630 nm (for the reference wavelength) was measured with the ELISA reader. All of the compounds were tested twice in the cell lines. The results expressed as IC_50_ (inhibitory concentration of 50%) were the averages of two determinations and were calculated by using the Bacus Laboratories Incorporated Slide Scanner (Bliss) software.

## 4. Conclusions

In summary, a novel series of dual diaryl urea and *N*-acylhydrazone derivatives were designed based on the hybrid pharmacophore concept. All the target compounds were synthesized and screened for their cytotoxicity against three human cancer cell lines (HL-60, A549 and MDA-MB-231) by standard MTT assays. The pharmacological results indicated that most compounds exhibited moderate to excellent activity. The preliminary SARs showed that both electron-withdrawing groups on Ar^1^ and 4- or 5-benzyloxyl groups on Ar^2^ are favorable for optimal cytotoxicity. Moreover, this encouraging research provides a valuable leading compound **2g** with IC_50_ values of 0.22, 0.34 and 0.41 μM against tested cell lines respectively, which were 3.8 to 22.5 times more active than the references sorafenib and PAC-1, and highlights the potential for further development of novel dual diaryl urea and *N*-acylhydrazone derivatives. Studies on the mechanism of action of these compounds are in progress and will be reported in the near future.
